# Synthesis of scaffold-free, three dimensional, osteogenic constructs following culture of skeletal osteoprogenitor cells on glass surfaces

**DOI:** 10.1016/j.bonr.2021.101143

**Published:** 2021-10-18

**Authors:** Latifa Alghfeli, Divyasree Parambath, Shaista Manzoor, Helmtrud I. Roach, Richard O.C. Oreffo, Ahmed T. El-Serafi

**Affiliations:** a Research Institute for Medical and Health Sciences, University of Sharjah, United Arab Emirates; b Bone and Joint Research Group, Institute of Developmental Sciences, University of Southampton, School of Medicine, UK; c Medical Biochemistry Department, Faculty of Medicine, Suez Canal University, Ismailia, Egypt; d Department of Biomedical and Clinical Sciences (BKV), Linköping University, Sweden

**Keywords:** 3D, three-dimensional, A/S, Alcian blue/Sirius red/Weigert's haematoxylin, ALP, Alkaline Phosphatase, BMP, bone morphogenic protein, BMSC, human bone marrow stromal, CSF, colony stimulating factor, Ct, Cycle threshold, EGF, epidermal growth factor, FC, Fetal bone cells, FCS, Fetal Calf Serum, FGF, fibroblast growth factor, FN1, fibronectin, gDNA, genomic DNA, GLI, GLI family zinc finger 1, HIPPIE, Human Integrated Protein Interaction Reference, iMSC, immortalized human bone marrow derived, mesenchymal stem cells, ITGA3, integrin A3, MMP, matrix metalloprotease, P/S, penicillin and streptomycin, R, receptor, TGF, β transforming growth factor beta, TGFBR2 transforming growth factor beta receptor 2 VDR, vitamin D receptor, vWF, von Willebrand factor, Osteoprogenitor cells, Pellets, 3D culture, Osteogenesis, Differentiation

## Abstract

**Background:**

Efficient differentiation of stem cells into three-dimensional (3D) osteogenic construct is still an unmet challenge. These constructs can be crucial for patients with bone defects due to congenital or traumatic reasons. The modulation of cell fate and function as a consequence of interaction with the physical and chemical properties of materials is well known.

**Methods:**

The current study has examined the osteogenic differentiation potential of human skeletal populations following culture on glass surfaces, as a monolayer, or in glass tubes as a pellet culture. The 3D prosperities were assessed morphometrically and the differentiation was evaluated through molecular characterization as well as matrix formation.

**Results:**

Early temporal expression of alkaline phosphatase expression of skeletal populations was observed following culture on glass surfaces. Skeletal populations seeded on glass tubes, adhered as a monolayer to the tube base and subsequently formed 3D pellets at the air -media interface. The pellets cultured on glass displayed 4.9 ± 1.3 times the weight and 2.9 ± 0.1 the diameter of their counterpart cultured in plastic tubes and displayed enhanced production of osteogenic matrix proteins, such a collagen I and osteonectin. The size and weight of the pellets correlated with surface area in contrast to cell numbers seeded. Global DNA methylation level was decreased in pellets cultured on glass. In contrast, gene expression analysis confirmed upregulation extracellular matrix proteins and osteogenesis-related growth factors.

**Conclusion:**

This simple approach to the culture of skeletal cells on glass tubes provides a scaffold-free, 3D construct platform for generating pellets enabling analysis and evaluation of tissue development and integration of multiple constructs with implications for tissue repair and regenerative application on scale-up.

## Graphical abstract

**Figure:**
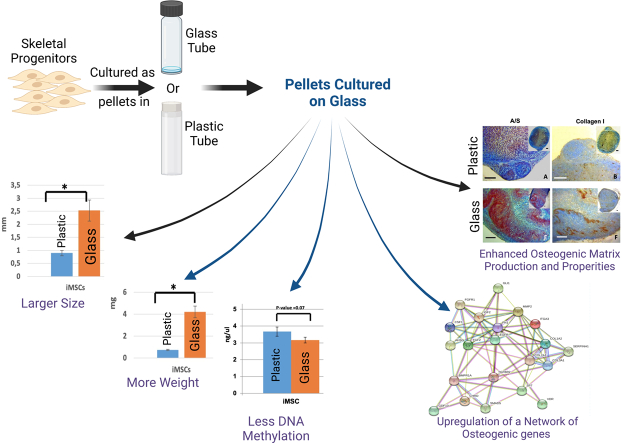

## 1 Background

The loss of skeletal tissue as a consequence of trauma, injury, disease or ageing can result in significant morbidity and socio-economic issues, exacerbated by an increasing ageing population combined with patient expectations for regenerative orthopaedic solutions. To date, current approaches to regenerate lost or damaged skeletal tissue hold a number of limitations including, but not limited to host-integration, scale, functionality and cost. To attempt addressing these issues, tissue engineering and regenerative medicine have emerged as approaches to tackle the unmet clinical need for bone augmentation and skeletal repair (Tang et al., 2016; Ambrosi et al., 2019). Approaches for bone defect repair include the use of artificial substitutes, while the use of bone grafts is typically, limited to spinal fusion, corrective osteotomy and maxillofacial reconstruction and can be associated with co-morbidity associated with the surgical wound site (Stiehler et al., 2008; Tuusa et al., 2007). Thus, strategies to incorporate and drive skeletal stem and osteoprogenitor cell differentiation into bone have garnered significant interest over the years, although, much remains to be resolved in the development of clinical scale functional bone constructs for routine patient application (Dawson et al., 2014; Pena Fernandez et al., 2020).

The cell responsible for bone formation, the osteoblast, is derived from an underlying source of skeletal stem cells present within the bone marrow stromal fraction. Friedenstein and co-workers first confirmed the presence of a fibroblast-like clonogenic precursor cells (colony-forming unit-fibroblastic/ CFU-F) in the tissue culture plastic (TCP) adherent non-hematopoietic fraction of bone marrow aspirate (Friedenstein et al., 1976). Furthermore, studies indicate that the multipotent stromal cells exist *in vivo* among the fibroblastic cells, known as pericytes, associated with the walls of the small blood vessels of the bone marrow and thus studies on the niche upon which these cells reside are informative (da Silva Meirelles et al., 2016; Yianni and Sharpe, 2019; Supakul et al., 2019). However, isolation of the skeletal stem cell population has proved difficult due to their low incidence, indeterminate morphology, and undefined phenotype. Nevertheless, strategies that can harness this fraction of renewable skeletal stem and progenitor cells offer significant potential in skeletal reparation. Fetal bone cells (FC) can be derived from the digestion and expansion of fetal bone tissue. We have shown previously, the ability of these cells to differentiate into the osteogenic and chondrogenic lineages as well as their ability for self-assembly into an osteochondral construct that resemble, in part, the cross section of the fetal bone construct (El-Serafi et al., 2011a; Mirmalek-Sani et al., 2006).

The differentiation of the multipotent cells is directed by a raft of different growth factors, hormones and chemical cues. In addition, cell interactions with the culture surface/microenvironment with activation of cell signalling cascades as a consequence of cell cytoskeletal–material interface interactions, which can be artificially organized at the nanoscale are known (Li et al., 2020). Thus, biomaterials are expected to play a crucial role in determining the stem cell fate. Several biomaterials were investigated in this context, including natural components of extracellular matrix, such as lyophilized collagen, fibronectin and decellularized bone matrix (Dzhoyashvili et al., 2015). On the other hand, synthetic biomaterials have the advantage of controlled synthesis, the possible incorporation of growth factors and avoiding any potential infectious agents (Prasadh and Wong, 2018; Lv et al., 2015). Classical examples include polylactic acid, polyglycolic acid and bio-ceramics (Cooke et al., 2010; Gao et al., 2017). These biomaterials can be formulated as a three-dimensional (3D) scaffold or as a culture surface. Ozawa and colleagues previously showed glass or glass ceramic could modulate cell adhesion and enhance the secretion of the alkaline phosphatase enzyme (Ozawa and Kasugai, 1996). However, to date, there has been a paucity of attention on the potential for the enhancement of osteogenic differentiation by glass in contrast to standard plastic culture materials. The current study investigated the effect of plain glass as a cell culture surface as a cost-effective, readily available and, critically, reusable material for skeletal cell differentiation on the differentiation of fetal bone cell and human bone marrow stromal (BMSC) populations as well as a human osteosarcoma osteoblast cell line, MG63 and an immortalized human bone marrow cell line. We hypothesized different response of the cells when cultured on glass as a consequence of the interaction between the cells and the culturing materials.

## 2 Materials and methods

Tissue culture reagents, media additives and all biochemical reagents were obtained from SigmaAldrich (Dorset, United Kingdom) unless otherwise stated. Fetal Calf Serum (FCS) was obtained from Invitrogen Ltd. (Paisley, United Kingdom). Graphical abstract was created with BioRender.com.

### 2.1 Cells

The study involved the use of primary cells from two sources, human adult bone marrow cells and fetal femur derived cells, as well as two cell lines, immortalized human bone marrow derived stem cells and MG63. Most of the experiments were verified with the four cell sources. The cell lines were particularly important when plenty of cells were needed, such as the morphometric evaluation of the cell pellets.

#### 2.1.1 Isolation and culture of human bone marrow derived skeletal cells

BMSCs were obtained from patients undergoing hip replacement surgery, with no history of haematological abnormalities, following the approval of the local ethics committee (Southampton and South West Hants Local Regulatory Ethics Committee LREC194/99/1) and obtaining an informed consent, as previously described (El-Serafi et al., 2011b). All experiments were performed in accordance with relevant guidelines and regulations. Cells were cultured under standard conditions, 37 °C and 5% CO_2_ in α-MEM (Lonza, Basel, Switzerland), supplemented with 10% FCS and 1% of 100 U/ml penicillin, 100 μg/ml streptomycin (P/S). All experiments were performed on cells established at passage 1.

#### 2.1.2 Isolation and culture of fetal femur derived cells

Human fetal tissue was obtained with informed and written consent from women undergoing termination of pregnancy procedure according to guidelines issued by the Polkinghorne Report. Ethical approval was obtained from the Southampton & South West Hampshire Local Research Ethics Committee (LREC 296100). All experiments were performed in accordance with relevant guidelines and regulations. Fetal femur samples, 7 to 12 weeks post conception (Gestational ages were estimated by stage according to the Carnegie classification or foot length) were isolated and skeletal muscle and connective tissues were removed from the femoral samples by rolling on filter paper and careful dissection. Femur samples were processed as whole or separated into epiphysial and diaphyseal sections by division at the metaphyseal regions. Femurs were plated into a well of a 6 well plate overnight in 2 ml α MEM containing collagenase B (5 mg/ml; Roche Diagnostics, Rotkreuz, Switzerland) at 37 °C as previously described (Mirmalek-Sani et al., 2006). Cell suspensions were passed through a 70 μm filter, centrifuged at 1100 rpm for four minutes and suspended in αMEM supplemented with 10% FCS and 1% P/S. Cells were maintained in monolayer culture under standard condition until 95% confluence prior to cell passage.

#### 2.1.3 Cell lines and culture conditions

Two cell lines were used; a) MG63, a cell line obtained originally form an osteosarcoma patient (ATCC, Virginia, United States), with known mesenchymal cell differentiation ability (Belka et al., 2019; Iaquinta et al., 2021; Wang et al., 2010; El-Serafi et al., 2019; Elsharkawi et al., 2019), cultured in DMEM, and b) immortalized human bone marrow derived, mesenchymal stem cells following hTERT transfection (iMSCs) purchased from Applied Biological Materials (British Columbia, Canada) and maintained in DMEM supplemented with 10% fetal calf serum and P/S, as well as 100 μM ascorbate-2-phosphate and 10 nM dexamethasone for osteogenic induction.

For three-dimensional pellet culture studies, cells in monolayer were detached from the plastic culture surface following incubation with collagenase type IV for 45 min followed by trypsinisation to achieve a single cell suspension (for the cell lines, collagenase step was not required). Cells were transferred at 5 × 10^5^ cells into 30 ml concave-end polystyrene universal tubes (Greiner Bio-One; Kremsmünster, Austria) in 1 ml of medium, centrifuged at 400 *g* for 10 min and then incubated at 37 °C under 5% CO_2_ (El-Serafi et al., 2011a). In the current studies, two types of 7-ml tubes with flat base were included; a) polystyrene (Thermo-Scientific, Massachusetts, United States) and glass (VWR, Pennsylvania, United States). The number of pellets was 3 to 5 per group. The pellets were cultured for three weeks in all experiments, except in the experiment with surface area of 90 mm as an extra week was necessary.

### 2.2 Alkaline phosphatase (ALP) histochemistry

Enhanced ALP activity can be considered as an early predictive marker for osteogenic differentiation of stem cells (Prins et al., 2014). Cells cultured in six well plates, with and without glass surface, were fixed in 90% ethanol and incubated in the dark, at 37 °C, with Napthol As-Mx Phosphate, mixed with 0.24 mg/ml Fast Violet B salt for 60 min. The reaction was stopped by rinsing samples with distilled water and left for air drying and ALP activity evidenced by red staining.

### 2.3 Histological studies

At the end of pellet culture study, the pellets were fixed in 4% (w/v) paraformaldehyde, followed by gradual dehydration and embedding in wax and sectioned at 7 um. Following dewaxing, sections were stained for the following;

#### 2.3.1 Alcian blue/Sirius red/Weigert's haematoxylin (A/S)

For general characterization of the matrix produced during the process of pellet formation, A/S stain was used, as described previously (El-Serafi et al., 2011a). The cell nucleus was visualized by Weigert's haematoxylin solution, which was applied to rehydrated samples for 10 min. After washing with water and acid alcohol to remove the excess stain, proteoglycans were stained with 8GX Alcian blue dissolved as 0.5% in 1% acetic acid, for 10 min. After rinsing with water, the slides were incubated with 1% molybdophosphoric acid for 20 min followed by one hour incubation in 0.1% Sirius red F3B solution for 45 min, in order to stain for collagen. Slides were rinsed thoroughly with water and dehydrated in reverse graded methanol into histoclear prior to mounting in dibutyl phthalate xylene (DPX).

#### 2.3.2 Immunocytochemistry

Immunocytochemistry was used to localize the expression of the key matrix proteins in the osteoid matrix (type I collagen and osteonectin) as well as the vascularization marker von Willebrand factor (vWF). Following removal of excess wax, endogenous peroxidase activity was quenched with 3% H_2_O_2_ (5 min) and non-specific binding blocked using 1% bovine serum albumin in PBS (30 min). The sections were incubated with relevant primary antibody overnight at 4 °C followed by biotinylated secondary antibody for an hour in the room temperature. The immune complex was visualized using the avidin–biotin method, linked to peroxidase and 3-amino-9-ethylcarbazole. Positive sections stained reddish-brown. Slides were counterstained with Alcian blue for one minute before a final wash and mounting in crystal mount. For negative controls, PBS was added instead of the primary antibody. All primary antibodies used were polyclonal raised in rabbits. Type I collagen, (LF67 (Fleischmajer et al., 1990)) and osteonectin (LF8 (Pacifici et al., 1990)) were gifts from Dr. Larry Fisher, NIH, Bethesda, USA. The antibody for vWF and the secondary antibody, anti- rabbit IgG biotin-conjugated, were purchased from Dako (Cambridgeshire, United Kingdom).

### 2.4 Gene expression analysis

In order to compare the change in gene expression of pellets cultured on both surfaces, RNA was extracted using the ‘RNeasy Mini Kit’ according to manufacturer's instructions (Qiagen, Hilden, Germany). The cell pellets were de-bulked by vigorous vortex with the lysing buffer and passed through a 0.6 mm gauge needle attached to an RNase free syringe 5 times, to ensure homogenization followed by a second vortex. The osteogenesis PCR arrays (Qiagen, Hilden, Germany) were used to validate gene expression for bone related growth factors and bone differentiation markers. Each array included a positive PCR control, genomic DNA contamination control, reverse transcription control in addition to 84 bone-related markers. RNA was reverse transcribed according to manufacturer's instructions after elimination for possible genomic DNA contamination. cDNA was added to RT2 qPCR Master Mix and aliquoted into different wells at a final volume of 25 μl. The mixture was incubated for 10 min at 95 °C followed by 40 cycles of 95 °C for 15 s and 60 °C for 1 min. Pellets cultured on plastic were set as reference and gene data presented as fold regulation with standard deviation. To investigate the correlation between differentially regulated genes, (supplementary material as Table S1) were uploaded to the ‘Gene Set Enrichment Analysis’ website (https://www.gsea-msigdb.org/gsea/index.jsp) and analysed against the pathway specific gene sets, using the ‘compute overlap’ function against the ‘GO gene sets’ with FDR q-value less than 0.05 (Subramanian et al., 2005; Mootha et al., 2003). Interactions between the gene protein products were investigated according to the Human Integrated Protein Interaction rEference (HIPPIE) (Alanis-Lobato et al., 2017). The interaction figures were prepared at STRING website (https://string-db.org/).

RNA extracted from MG63 pellets was reverse transcribed into cDNA using TruScript First Strand cDNA Synthesis Kit according to the recommended thermal protocol (Norgen Biotek). Then, 10 μl of sybr green based GoTaq® qPCR Master Mix (Promega) was used with 100 ng cDNA in a reaction volume of 20 μl in the thermal cycler Rotorgene Q (Qiagen). The cycling conditions involved initial heating at 95 °C, followed by 45 cycles of denaturation 95 °C and annealing/ extension at 60 °C for 60 s. Melt curves were obtained at the end of each run for quality control. All reactions were run in triplicate. A negative control was included in each run and contained ultrapure water instead of the cDNA. Ct value for each sample was normalized to GAPDH, an endogenous housekeeping gene, and fold expression levels for each target gene calculated using the delta-delta Ct (cross-over threshold) method (Livak and Schmittgen, 2001). The reference for each gene was the expression in the pellets cultured on plastic.

### 2.5 Global DNA methylation analysis

The level of DNA methylation was investigated as decrease methylation has been associated with enhanced osteogenic differentiation of stem cells (El-Serafi et al., 2011b). DNA was extracted using the ‘All in One Purification Kit’ according to manufacturer's instructions (Norgen Biotek, Thorold, Canada). Following the lysis step, the lysate was loaded into the genomic DNA (gDNA) isolation column and the eluted gDNA was quantified using the Nano drop 2000 spectrophotometer (Thermo-Scientific, Massachusetts, United States). Methylated DNA quantification kit (Abcam, Cambridge, UK), an enzyme-linked immunosorbent assay, was used for this study. The capturing antibody for 5-methylcytosine was incubated with 50 ng DNA and, following the steps in the manufacturer's manual, the developed colour was read spectrophotometrically at 450 nm. The standard curve showed R2 = 0.9646.

### 2.6 Statistical analysis

The data was analysed using the Data Analysis ToolPak (Microsoft® Excel, Microsoft® Office 365). Statistical significance was evaluated using Student's *t*-test with the p-value threshold set at 0.05. For the PCR Array data, the cycle threshold figures was uploaded to the online tool ‘RT^2^ Profiler PCR Data Analysis’ (https://dataanalysis2.qiagen.com/pcr), according to set templates and the fold expression was calculated using the delta-delta Ct approach (Livak and Schmittgen, 2001).

## 3 Results

### 3.1 MG63 cells cultured on glass slides express alkaline phosphatase earlier than cells maintained on tissue culture plastic

**Fig. 1:**
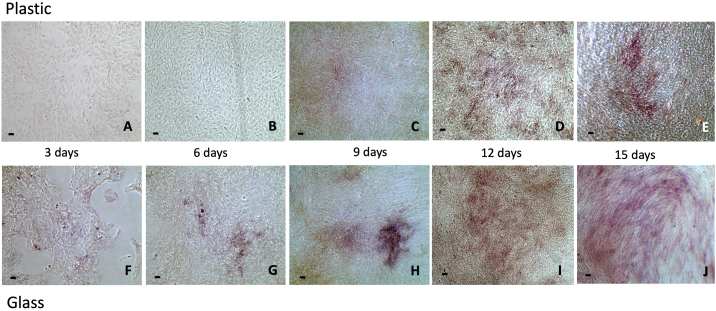
The activity of ALP commenced on day 3 for MG63 cells cultured on glass (F) with increasing intensity until day 15 (G-J). For the cells cultured on plastic, the activity commenced on day 9 (C) with increasing intensity on day 12 (D) and day 15 (E). The ALP intensity was enhanced on glass compared to plastic over all time points examined. Scale bars = 100 μm.

Alkaline phosphatase activity, a surrogate osteoblast marker was examined in MG63 cells, following monolayer cell culture in six well plates with and without glass surfaces, in osteogenic media and at different time points. The ALP staining, red in colour, reflects the level of enzyme activity. On glass slides, MG63 cells commenced the expression of ALP activity as early as day 3 of culture with enhanced expression noted through to the end of culture on day 15 (Fig. 1). On tissue culture plastic, ALP expression was not observed until day 9 and, the intensity of ALP expression was reduced in comparison to cells cultured on glass slides at the various time points examined, indicating enhancement of alkaline phosphatase expression and differentiation on glass slides.

### 3.2 Skeletal populations culture as pellets on glass retain self-assembly conformation with enhanced osteogenic differentiation

**Fig. S1:**
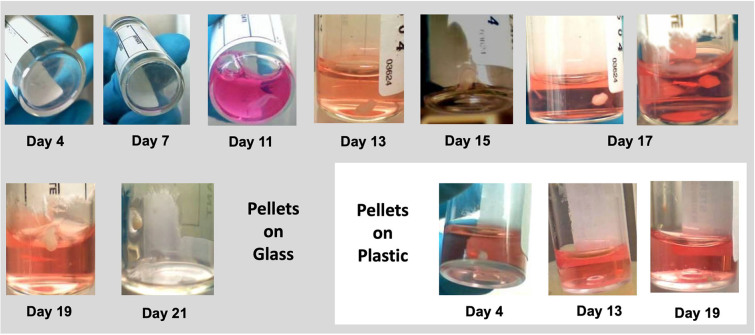
Fetal femur derived cells, seeded in glass tubes, adhered to the tube base as a monolayer for 7 days, and subsequently peeled and rolled to form a pellet by day 13. On day 15, the pellet migrated up the tube sidewall, a process that was noted to continue until day 17, when a band of cells emerged from the pellet, adhered to about two thirds of the inner tube circumference to end in a thick band at the media-air interface. On day 19, the whole pellet was observed at the position of the thick band, covered with thin film of media. Side-view on day 21 demonstrated the 3D configuration of the pellet, with position relative to the tube base and size relative to the tube. Cells cultured in plastic tubes retained their size and configuration throughout the culture (n = 5).

The sequence of events involved in the pellet formation varies between the plastic and glass tubes. Examination of BMSCs, fetal populations, MG63 and iMSCs on glass and plastic tubes indicated retention of cell pellet formation and enhanced osteogenesis on glass substrates (Fig. S1). Pellets maintained on plastic displayed a spherical conformation as early as day four and retained their shape and size over 3 weeks of culture. BMSCs seeded in glass tubes displayed a distinct morphological transition with cells adherent to the glass substrate, as a monolayer, which increased in density up to day 7. The cell sheet subsequently peeled to form a rolled aggregate by day 12 ± 2 days. The pellet was noted to migrate across the tube over 48 h and adhere to the sidewall by day 13 ± 1 day and subsequently pellets typically migrated up the glass tube wall. Cells were observed to proliferate from the pellet and to extend gradually over two thirds of the inner circumference of the tube forming a thick layer of cells across the liquid-air interface. Typically, after 48 h, the pellet merged with the band of cells at the liquid-air interface. The pellet, on close observation, remained covered by a thin film of media until the end of the culture period.

Cultures of iMSCs displayed a comparable sequence of events as for the MG63 cultures, although growth of the osteosarcoma MG63 cells was observed to be enhanced in comparison to BMSCs and iMSCs (Table 1). Critically, pellets were observed to form in all tubes with BMSC and iMSCs, while, 20% of the tubes containing MG63 cells failed to form a final pellet.

**Table 1:** Timeline (in days) for pellet formation in Fetal cells (FC), MG63 and iMSCs.

	FC	iMSCs	MG63
High density monolayer	7	6	5
Peripheral detachment	12	12	6
Migration to tube side wall	13	15	12
Migration to the air-media interface	15	16	14
Pellet at the air-media interface	19	20	18

### 3.3 Matrix characterization of cell pellets

**Fig. 2:**
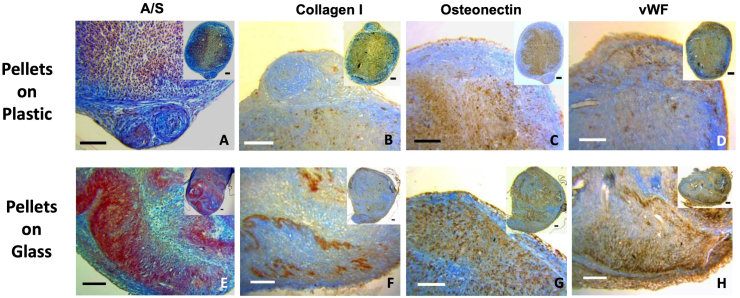
A/S staining of FC pellets showed negligible matrix formation when cultured in plastic tubes (A), in contrast, marked enhancement of osteogenic matrix was obtained in self-assembled pellets cultured in glass tubes (E). Immunostaining for collagen I, osteonectin and vWF showed enhancement of the studied markers for the pellets cultured in glass tubes (F, G and H) in comparison to plastic (B, C and D). Interestingly, while collagen I followed the Sirius red staining, vWF seemed to be overlaid on the collagen level (H). Scale bars = 100 μm.

**Fig. S2:**
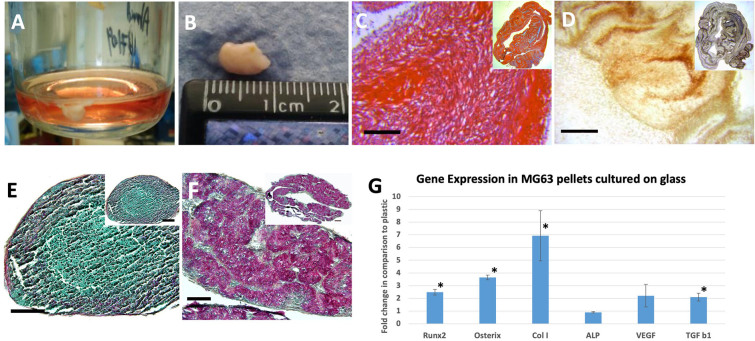
Characterisation of fetal femur derived cells seeded in a 90mm diameter bottle (A-D). The final pellet is shown in (A). The pellet reached 9 mm in length (B). The matrix displayed intense Sirius red (C) and collagen I staining (D). Characterisation of MG63 pellets on plastic (E) and glass (F) stained with Alcian blue and Sirius red, as well as the relative gene expression of pellets cultured on glass, in comparison to pellets cultured on plastic. * p<0.05.

The matrix production by fetal cells pellets has been assessed with histological (A/S) staining to observe proteoglycan (blue), and collagen (red). Histological characterization of pellets cultured on plastic showed compact spherical conformation. On glass, the osteoid matrix folded into sheets (Fig. 2). While limited osteoid formation was observed on plastic substrates, intense matrix deposition was observed on fetal femur derived cell pellets grown on glass vials with chondrogenic matrix, evidenced by Sirius red and alcian blue staining, present within the pellet construct (Fig. 2A and E). Further characterization with immunohistochemistry showed the presence of bone matrix (type I collagen and osteonectin) and vascular factor (vWF). Pellets cultured on glass showed enhanced deposition of type I collagen (Fig. 2B, F) and osteonectin (Fig. 2C and G) aligned to Sirius red staining. vWF was markedly enriched in relation to areas of osteogenic matrix (Fig. 2D and H). The expression of the studied osteogenic markers was enhanced on pellets cultured on glass vials in comparison to pellets cultured on cell culture plastic alone (Fig. 2A–D compared to Fig. 2E–H). The pattern of matrix deposition indicated osteogenic differentiation in relation to the cells adherent to the glass. Interestingly, endothelial marker expression (vWF) was observed adjacent to this layer and chondrogenic differentiation in the cells trapped between the osteogenic folds. Alcian blue/Sirius red staining for osteogenic matrix in MG63 pellets displayed a similar pattern of enhanced osteogenic matrix formation (Fig. S2E, F).

### 3.4 Morphometric analysis of the cell pellets cultured on glass surfaces

**Fig. 3:**
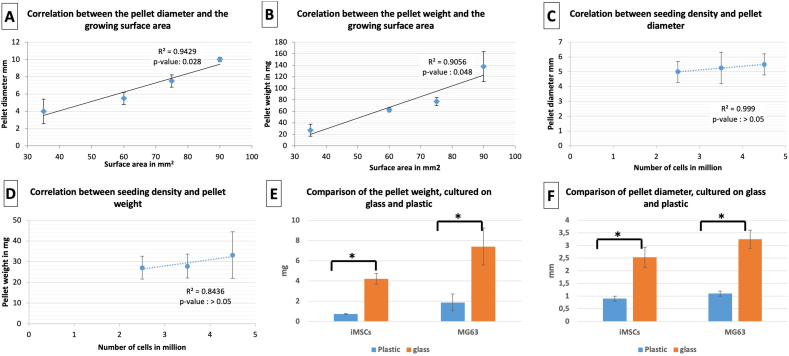
After three weeks in culture, the pellet diameter and weight, were positively correlated with the surface area of the glass tube base (A and B). Pellet diameter and weight did not correlate with MG63 seeding density (C and D). The pellet weight and diameter were significantly higher on glass in comparison to plastic, for both MG63 and iMSCs (E and F).

As the difference between the pellets size was clearly observed, the size and weight were compared using a simple morphometric approach that depends on the measurement of the diameter and weight of the pellet. The initial phase of formation of such large pellets was the adherence to the tube base, the correlation between pellet size and the surface area of the tube base was examined (n = 4). MG63 cells were seeded in glass vials of different sizes with cell seeding determined by the surface area of the vial base (established at 10^6^ cells/mm^2^). Pellet size and wet weight positively correlated to the glass vial surface area (Fig. 3A, B). With surface area of 90 mm, the pellets displayed an average diameter of 9.5 ± 0.75 mm and weight of 137.65 ± 18.4 mg (Fig. 3C). Different seeding densities (2.5, 3.5 and 4.5 × 10^6^ cells) within 50 ml glass vials of the same diameter (Fig. 3D), resulted in cultures with comparable morphometric characteristics. There were no statistical differences observed in three studies, indicating cell adherence to the glass surface enhanced the pellet formation based on surface area. The self-assembled pellets cultured on glass contained 4.9 ± 1.3 times the weight of their counterpart cultured in plastic tubes and displayed a diameter 2.9 ± 0.1 times larger (Fig. 3E and F). Interestingly a pilot study using fetal femur derived cells seeded on a glass vial with a base diameter of 90 mm and cultured for 28 days, displayed comparable morphology and characteristics (Fig. S2). The pellet long axis reached 9 mm. Histological staining showed intense Sirius red in A/S staining, indicative of collagenous proteins and subsequently confirmed to be predominantly collagen type I by immunohistochemistry (Fig. S2A–D).

### 3.5 Differential gene expression in iMSCs grown on glass and plastic surface by osteogenic PCR Arrays

**Fig. 4:**
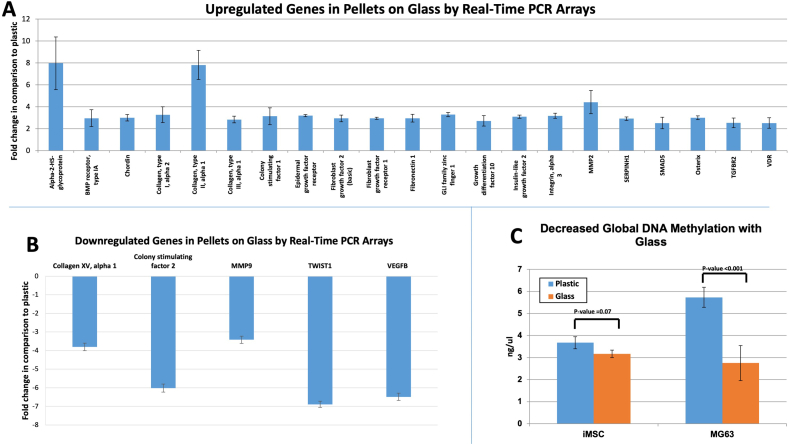
(A) Change in gene expression in pellets cultured on glass and plastic, with the latter as the reference. Out of the 84 markers studied in the PCR arrays, 21 genes were significantly upregulated including; 1) cell surface receptors, such as vitamin D receptor (VDR), transforming growth factor beta receptor 2 (TGFBR2), fibroblast growth factor receptor 1, epidermal growth factor receptor and bone morphogenic protein receptor 1A; b) extracellular matrix proteins, including collagen type Iα2, IIα1, IIIα1, fibronectin, their cell mediator protein, Integrin subunit α3 and the modulating enzyme matrix metalloprotease (MMP) 2; c) signalling molecules including, osterix, growth differentiation factor 10, fibroblast growth factor 2, colony Stimulating factor 1, chordin, SMAD5, SEPRINH1, somatomedin A, GLI family zinc finger 1 and alpha 2 HS glycoprotein. Five genes were downregulated, including collagen type XV α1, colony stimulating factor 2, MMP-9, twist1 and vascular endothelial growth factor. (B) Global DNA methylation level showed decreased levels of DNA methylation with MG63 seeded in glass tubes and a comparable observation in iMSCs samples.

**Fig. 5:**
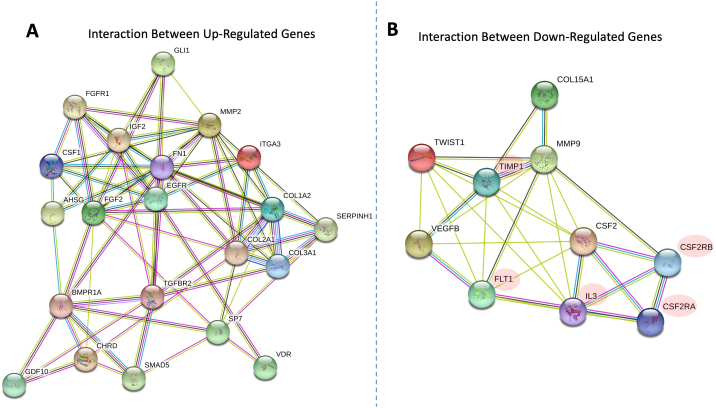
(A) The interaction between the upregulated genes showed an central role for fibronectin with direct connections to the matrix. (B) The relation between downregulated genes (with green background) showed connection with expected intermediates (white background), according to HIPPIE – Human Scored Interactions. The figures were prepared at STRING website (https://string-db.org/).

In order to characterize the changes in gene expression related to culturing the cells on the two substrates, an 84 gene panel array was selected. This PCR array included markers for bone mineral metabolism, growth factors and genes mediating osteogenesis and related cell growth, proliferation, and differentiation processes. From the 84 genes examined, 20 genes were observed to be significantly upregulated in the pellets cultured on glass in comparison to those on plastic (Fig. 4A), including; a) a group of cell surface receptors, such as vitamin D receptor (VDR; 2.5 ± 0.5 fold), transforming growth factor beta (TGF- β) receptor 2 (TGFBR2; 2.5 ± 0.5 fold), fibroblast growth factor receptor 1 (3.0 ± 0.3 fold), epidermal growth factor receptor (3.2 ± 0.3 fold) and bone morphogenic protein receptor 1A (3.0 ± 0.8 fold); b) extracellular matrix proteins, including collagen type Iα2 (3.3 ± 0.7 fold), IIα1 (7.8 ± 1.3 fold), IIIα1 (2.8 ± 0.3 fold), fibronectin (3.0 ± 0.4 fold), their cell mediator protein, Integrin subunit α3 (3.2 ± 0.2 fold) and the modulating enzyme matrix metalloprotease (MMP) 2 (4.4 ± 1.1 fold); c) growth factors, transcription factors and intracellular signalling molecules including, osterix or SP7 (3.0 ± 0.2 fold), growth differentiation factor 10 (2.7 ± 0.5 fold), fibroblast growth factor 2 (3.0 ± 0.3 fold), colony Stimulating factor 1 (3.1 ± 0.8 fold), chordin (3.0 ± 0.3 fold), SMAD5 (2.5 ± 0.5 fold), SEPRINH1 (2.9 ± 0.2 fold), somatomedin A (3.1 ± 0.2 fold), GLI family zinc finger 1 (GLI; 3.3 ± 0.2 fold) as well as alpha 2 HS glycoprotein (8 ± 2.4 fold). The upregulated genes, as a consequence of cell culture on glass, showed overlap with 9 sets of genes within the ‘Gene Set Enrichment Analysis’ website with the first hit with the skeletal system development set. Other upregulated sets included ossification, regulation of cell differentiation, as well as multiple signalling and response to growth factor pathways, as shown in Table 2. The protein-protein interaction of those gene products (Fig. 5A) showed a central role of fibronectin that can enhance multiple cell surface receptors and consequently the intracellular signalling cascade, which resulted in enhancing differentiation and secretion of relevant extracellular proteins. In addition, 5 genes were downregulated (Figs. 4B and 5B), including collagen type XV α1 (−3.8 ± 0.2 fold), colony stimulating factor 2 (−6.0 ± 0.2 fold), MMP-9 (−3.4 ± 0.2 fold), twist1 (−6.9 ± 0.2 fold) and vascular endothelial growth factor (−6.5 ± 0.2 fold). The corresponding gene sets included cellular proliferation, macrophage differentiation as well as collagen catabolic process (Table 2).

**Table 2:** Gene sets overlap with the ‘gene set enrichment analysis’ database.

	Upregulated gene set name	No of genes	p-value	FDR q-value
1	Go skeletal system development	15	1.83 e-24	1.88 e-20
2	Go ossification	13	2.1 e-21	1.08 e-17
3	Go enzyme linked receptor protein signalling pathway	15	1.29 e-19	4.41 e-16
4	Go tube development	15	3.04 e-19	7.8 e-16
5	Go cellular response to endogenous stimulus	15	6.23 e-18	1.28 e-14
5	Go animal organ morphogenesis	14	1.01 e-17	1.73 e-14
6	Go regulation of cell differentiation	16	1.44 e-17	2.12 e-14
7	Go response to endogenous stimulus	15	7.46 e-17	9.58 e-14
8	Go response to growth factor	12	3.88 e-16	4.43 e-13
9	Go embryo development	13	5.2 e-16	5.34 e-13


	Downregulated gene set name	No of genes	p-value	FDR q-value
1	Go positive regulation of cell population proliferation	4	1.74 e^−6^	1.78 e^−2^
2	Go anatomical structure formation involved in morphogenesis	4	3.93 e^−6^	2.02 e^−2^
3	Go collagen catabolic process	2	1.34 e^−5^	2.57 e^−2^
4	Go macrophage differentiation	2	1.34 e^−5^	2.57 e^−2^
5	Go regulation of phosphorylation	4	1.45 e^−5^	2.57 e^−2^
6	Go regulation of cell population proliferation	4	1.81 e^−5^	2.57 e^−2^
7	Go positive regulation of multicellular organismal process	4	2.2 e^−5^	2.57 e^−2^
8	Go positive regulation of dna binding	2	2.2 e^−5^	2.57 e^−2^
9	Go regulation of phosphorus metabolic process	4	2.27 e^−5^	2.57 e^−2^
10	Go regulation of protein modification process	4	2.5 e^−5^	2.57 e^−2^

The gene expression of cell pellets of MG63 showed upregulation of the osteogenic master gene, Runx-2, by 2.5 ± 0.2 fold for pellets maintained on glass in comparison to plastic. The transcription factor osterix was upregulated 3.7 ± 0.2 times and the growth factor TGF-β1 was 2.1 ± 0.3 folds. The expression of collagen I, was upregulated 6.9 ± 2.0 times. Vascular endothelial growth factor was observed to be upregulated 2.2 ± 0.9 fold, although this failed to reach statistical significance. The enzyme alkaline phosphatase activity showed no differences between the two groups, at the time points examined (Fig. S2G).

### 3.6 Evaluation of global DNA methylation

DNA demethylation can be associated with enhanced osteogenesis. DNA methylation was evaluated for pellets cultured on glass and plastic (Fig. 4C). There was a significant decrease (P < 0.001) in the methylation level for MG63 pellets cultured on glass (5.7 ± 0.5 ng/ul) in comparison to plastic (2.8 ± 0.8 ng/ul). A similar trend was observed in iMSC for glass (3.2 ± 0.2 ng/ul) and plastic (3.7 ± 0.3 ng/ul) although this did not reach the statistical significance (p = 0.07).

## 4 Discussion

There remains a significant unmet need for simple strategies to generate significant amounts of new skeletal tissue. In this study, we compared two models of 3D tissue engineered osteoid organoids. The first model was based on the standard pellet culture, in plastic tubes, which followed the previously described approach (12). This model is based on accumulation of cells, in the form of a pellet, that would transform into a sphere within a few days. The cells would display differentiation markers and matrix development dependent on culture media employed (Kim and Adachi, 2021; Kim and Adachi, 2019; Knuth et al., 2017). Furthermore, the current studies demonstrate that the changes in gene expression pattern is enhanced when the cells are cultured in three dimensional culture systems, in comparison to monolayer culture (El-Serafi et al., 2019). This innovative model is based on the observation that the same number of the cells would preferentially adhere to the glass surface to form a monolayer that followed a certain cascade of events including rolling of the monolayer to one of the sides of the glass tube, attachment to the side wall and migration of the self-assembled construct to the air-media interface. These constructs were larger in size, richer in matrix and associated with upregulation of several osteogenic lineage genes.

Self-assembly is a modern approach for tissue engineering that seeks to enhance the maturity of differentiated cells (El-Serafi et al., 2011a; El-Serafi et al., 2017). This approach was defined as a ‘semi-natural’ process that mimicked the embryonic organogenesis through guided differentiation milieu (Larouche et al., 2009) and widely adopted including the application, *in-vivo,* with injectable hydrogels. In this case, the thermodynamic properties and the interaction with the surrounding environment can guide the assembly process (Lee et al., 2017). Chondrogenic self-assembly has been described in non-adherent agarose wells. The construct properties are based on the cell type present. Cord blood derived cells were superior to bone marrow cells, in terms of the construct size and properties (White et al., 2018). In 2006, Hu and Athanasiou cultured bovine chondrocytes at high density on agarose (5.5 × 10^6^ cells in 300 μl media). Cells remained detached from the plastic surface and formed a cohesive nodule after 24 h (Hu and Athanasiou, 2006). Over 12 weeks, the nodule appeared smooth and flat while the comparable construct cultured on plastic was contorted into folded layers. In addition, the agarose-cultured nodule was significantly higher in collagen and proteoglycan content at different time points. The authors argued that the self-assembling constructs would avoid the drawbacks of scaffolds. In the current system, the adhesion of cells to the glass over time, enhanced matrix formation. Similarly, chondrocyte maturation during bone formation, is associated with the interaction with the developed scaffold and available reactive oxygen species and antioxidants (Nugud et al., 2018). *In-vitro*, pellet culture is the classic example of scaffold-free cell culture. The cells are directed by centrifugation to aggregate, enhance interaction, stimulate extracellular matrix protein production and cell differentiation, although, the pellets may have mechanical and geometrical limitations (Lee et al., 2017).

The migration of the pellets toward the air-medium interface has not been described before, to the best of our knowledge. Bryant et al. described the migration of leucocytes against gravity on glass slides (Bryant et al., 1966). This process was preceded by centrifugation of the whole blood and mounting on glass slides, where the migration process continued for 12 h. The authors found that leukocyte migration was not affected by changes in the plasma pH, electrolytes or glucose level. Alternatively, the migration was enhanced by increasing the temperature from 25 °C to 40 °C and both the adhesion to glass and the migration were markedly inhibited by heating the cell-free plasma at 56 °C for 30 min. The authors concluded that certain heat labile factors were important in the adhesion and migration of the cells. Annabi et al. studied the effect of hypoxia on murine BMSCs cultured in Matrigel. The cells migrated in the Matrigel to form a capillary-like network after 4 h of culture, 1% oxygen (Annabi et al., 2003). The membrane-type 1 metalloproteinase (MMP) 1 was markedly induced in the cells and blocking the catalytic domain of MMP1 diminished the migration ability of the cells. Similarly, MMP-2 was upregulated in our gene expression study.

Fetal skeletal cells were characterized as easy to isolate following digestion of the human fetal femur with collagenase. This cell population is at an early stage of commitment, maintaining their multipotential capacity. Following isolation, fetal skeletal cells can be easily expanded in culture and respond efficiently to the three lineage differentiation assays; *i.e.* osteogenic, chondrogenic and adipogenic lineages (Mirmalek-Sani et al., 2006). We have previously reported a 3D osteogenic model based on fetal skeletal cells (El-Serafi et al., 2011a). The cells, upon culture as pellets in osteogenic media, were able to self-organise and generate three layers that consisted of a bone shell that enclosed a chondrogenic matrix surrounded by a fibrous layer. Such an arrangement would reflect, in part, the natural assembly of fetal tissue. In the skin, Jean et al. built a construct by growing fibroblasts and keratinocytes as monolayers and then adding two layers of fibroblasts in the top of each other with seeded keratinocytes on the top. The structure was moved to the media air interface and showed the histological features of skin multilayers (Jean et al., 2011). The current study reported self-assembled pellets with enhanced morphometric parameters in comparison to classical pellets. The reasons may include enhanced cell number as evidenced by the thickening of the monolayer prior to the rolling phase, the presence of empty spaces during rolling, which was filled later by the matrix, as well as the material effect that enhanced differentiation and matrix secretion. In pellet cultures, the presence of satellite small pellets is a common observation as some cells fail to integrate into the developing pellet, comparable to the observation of some cells to adhere to scaffolds upon seeding. In the self-assembled pellets, the cells formed a monolayer that was folded into a 3D configuration with minimal cell loss. Such an observation could prove useful in translation to 3D regeneration. The consistency of the findings between the studied cell types, does not only support the results, but also confirm the steadiness of the findings among osteoprogenitor cells that could be at different stages of differentiation.

The diverse effects of the materials examined, as well as the cell contact surface on cell differentiation were expected. Glass has been historically used for cell culture and the initial plastic containers for this purpose were coated with glass to support cell attachment (Barker and LaRocca, 1994). Furthermore, the nanopatterning of the plastic can either enhance maintenance of the native state or enhancement of differentiation of the cell through integrin-dependent signalling pathway (Dalby et al., 2014). The latter is expected to be involved in the present model as shown by the upregulation in integrin A3 (ITGA3). In this model, glass provides a platform for osteogenic development similar to the chondrogenic anlage during fetal life. This anlage is essential for the recruitment of the osteoprogenitors which would differentiate and produce components of the bone matrix (Hall and Miyake, 2000; Sandell and Adler, 1999). In the current system, the initial adhesion between the cells and the glass surface may facilitate differentiation of the cells and matrix formation as triggered by the osteogenic inducing media. Borosilicate, the main constituent of the glass tubes, has been shown to enhance the expression of the osteogenic differentiation markers in BMSCs when cultured on the top of the cells as a powder. Furthermore, borosilicate forms a bone-like apatite crystals when immersed in a simulated body fluid *in vitro* (Fernandes et al., 2016). No differences were observed between the pellets cultured on the flat base tubes and the conical base tubes used in previous experiments (data not shown), indicating that the material of the tubes was more important than the configuration of the tube itself. The presence of vWF positive cells could indicate the differentiation of stem cells into endothelial cells, especially with the localization along the osteogenic matrix. This indicator of vascularization is crucial for early bone regeneration and has been described previously as a differentiation target of BMSC (Li et al., 2018).

Rat-derived BMSCs display enhanced ALP activity when cultured over discs made of a mixture of glass and ceramic, in comparison to titanium discs (Ozawa and Kasugai, 1996). These results were explained by the roughness of the surface as well as the adsorption of certain chemicals in the media that could alter the microenvironment around the cells. Keselowsky et al. (2005) investigated surfaces coated with fibronectin as well as chemically modified fibronectin; *i.e.* two different ligands for the cell surface integrins. Ligand terminal contained –OH or –NH2, was enhanced ALP activity, up regulation of osteogenic genes and matrix mineralization in comparison to the terminals with –COOH or –CH3. Integrin blocking antibody inhibited the mineralization with –OH or –NH2, while functioning antibody stimulated the matrix mineralization with –COOH and –CH3. Thus, the surface effect on the cells could be transmitted through cell surface integrins. In our analysis of upregulated genes, fibronectin (FN1) plays a central position in protein-protein interaction influenced by ITGA3 which, in turn, can enhance collagen 1 A2 expression, while FN1 can directly enhance the production of collagen 2 A1 and MMP-2. These genes explain, in part, the findings of enhancement and remodelling of osteogenic matrix deposition. Furthermore, FN1 induced the expression of a number of growth factor receptors (R), including transforming growth factor beta (TGFß) R2 as well as bone morphogenic protein (BMP) R1A, epidermal growth factor (EGF) R and fibroblast growth factor (FGF) R. The TGFß-BMP axis is crucial for mesenchymal cell transition into osteoblasts, especially through BMPR1A. Overexpression of this receptor induced osteoblastic phenotype in C2C12 cells, even in the absence of a receptor ligand. The role of TGFßR2 involves calcium homeostasis and bone metabolism, which could indicate a next step of maturity (Rahman et al., 2015). Smad 5, also upregulated in the current study, plays a role in regulation of the crosstalk between BMP and FGF in endochondral bone formation (Retting et al., 2009). Thus, the FGF pathway links through Smad 5 and through the upregulation of FGFR1. Inactivation of FGFR1 in osteoprogenitors, can be associated with delayed osteoblast differentiation. The ligand, FGF2, can enhance bone formation at the basal level, as well as under the influence of parathormone (Marie et al., 2012). GLI1, another upregulated transcription factor upstream of FN1, is present in mesenchymal and osteoprogenitors but not mature osteoblasts (Shi et al., 2017). In addition, IGF2 can enhance the osteogenic induction of BMP9 on MSCs through Smad 5 as well as the expression of alkaline phosphatase and matrix mineralization (Chen et al., 2010). EGFR was upregulated on glass in comparison to plastic. EGF has been shown to enhance the differentiation of dental pulp stem cells into the osteogenic lineage, when added to the basic osteogenic media (Del Angel-Mosqueda et al., 2015). Furthermore, EGF can enhance the BMP-9 induction of early and late osteogenic markers, as well as ectopic bone formation in mouse model. BMP-9 can induce EGFR through the Smad 1, 5 and 8 (Liu et al., 2013). Osterix or SP7, is one of the key transcription factors required for the differentiation of stem cells into osteoblasts. VDR is a downstream gene that has an osterix-responsive element on its promoter crucially upregulated during the differentiation process (Zhang et al., 2011). Interestingly, both markers were upregulated on glass in comparison to plastic.

The downregulated genes included; i) TWIST1, a negative regulator of bone formation and development through antagonizing the effect of Runx-2 (Bialek et al., 2004); ii) matrix metalloproteinase 9 (MMP-9) involved in the organization of extracellular matrix and knocking it enhances the density of bone trabeculae (Nyman et al., 2011); iii) colony stimulating factor 2 (CSF-2) subject to topographic modulation in mesenchymal stem cells (Hyzy et al., 2017); iv) collagen type 15, a facet type of collagen that is mainly present in the placenta, adipose tissue and other soft tissues (Liu et al., 2017). Gene expression for MG63 pellets cultured on glass and on plastic displayed a similar trend for the tested markers. The decrease of DNA methylation level is in agreement with our previous studies, which correlated with the application of DNA methylation inhibition to reduce cell differentiation and osteogenic differentiation in association with culture conditions (Elsharkawi et al., 2019; El-Serafi, 2012; El-Serafi et al., 2011c). High expression of integrins can be associated with DNA hypomethylation and activation of several genes (Carpenter et al., 2017; Chen et al., 2009).

The interaction between the cells and the substrate, at the molecular level, will be an interesting topic for further elucidation. The physicochemical characteristics of the culturing surface would affect the cell adhesion and intracellular signalling cascade. The nanotopography of plastic has been shown to affect the cell adhesion and differentiation (Dalby et al., 2014). On the other hand, glass can adsorb several components from the surrounding milieu which allow the precipitation of hydroxyapatite crystals, a key component of the osteogenic matrix (Zhang et al., 2002).

## 5 Conclusion

In conclusion, the current studies indicate the potential of different skeletal cell populations to self-assemble into osteogenic constructs, larger in size and richer in matrix than the classical pellet model systems. This ‘on-glass’ model offers an approach to address the often limited number of cells that can be obtained from a patient. The folding cell tissue pattern offers a microenvironment for neovascularization and enhanced integration and channels through which nutrients and oxygen can penetrate to the centre of the construct *in vitro* and following *in vivo* transplantation. The ability to combine multiple pellets to place within a repair area offers an intriguing prospect of a physiological engineered, scaffold free bone constructs. The characterization of the ‘on-glass’ model in comparison to the wide range of available scaffolds may clarify the efficiency of our new model in terms of the 3D size that can be obtained with a certain number of cells. In addition, the comparison of their osteogenic differentiation potential would help in identifying the best construct for clinical use in the future, which can be considered as a limitation in this study. The other limitation was the absence of *in vivo* data. The sustainability of the pellets cultured on glass and the integration in a bone defect model would be interesting to evaluate in the future. The current studies offer an approach to examine cell differentiation in a spatial cost-effective, simple model, with significant potential for a novel model of 3D cell culture for skeletal cell differentiation and matrix formation with application in skeletal physiology.

The following are the supplementary data related to this article.

Table S1 List of the differentially regulated genes in the PCR arrays Table S2

## CRediT authorship contribution statement

**Latifa Alghfeli:** Investigation, Writing – original draft. **Divyasree Parambath:** Investigation, Writing – original draft. **Shaista Manzoor:** Investigation, Validation. **Helmtrud I. Roach:** Conceptualization, Validation. **Richard O.C. Oreffo:** Conceptualization, Validation, Writing – review & editing, Funding acquisition. **Ahmed T. El-Serafi:** Conceptualization, Validation, Formal analysis, Project administration, Writing – review & editing, Funding acquisition.

## Declaration of competing interest

None.
