# Supplementary material for: Synthesis of scaffold-free, three dimensional, osteogenic constructs following culture of skeletal osteoprogenitor cells on glass surfaces
Source: Bone Rep. 2021 Oct 18;15:101143. doi: 10.1016/j.bonr.2021.101143 (PMC8554168; doi:10.1016/j.bonr.2021.101143)
Supplement: Table S1 — List of the differentially regulated genes in the PCR arrays [file mmc3.docx]

Table (S1). List of the differentially regulated genes in the PCR arrays

| **Gene** | **Gene Description** | **Fold regulation** | **SD** |
| --- | --- | --- | --- |
| AHSG | Alpha-2-HS-glycoprotein | 7.97 | 2.39 |
| BMPR1A | BMP receptor, type IA | 2.96 | 0.77 |
| CHRD | Chordin | 3.00 | 0.29 |
| COL15A1 | Collagen, type XV, alpha 1 | -3.79 | 0.20 |
| COL1A2 | Collagen, type I, alpha 2 | 3.28 | 0.71 |
| COL2A1 | Collagen, type II, alpha 1 | 7.83 | 1.34 |
| COL3A1 | Collagen, type III, alpha 1 | 2.84 | 0.32 |
| CSF1 | Colony stimulating factor 1 | 3.14 | 0.77 |
| CSF2 | Colony stimulating factor 2 | -6.02 | 0.22 |
| EGFR | EGFR Epidermal growth factor receptor | 3.20 | 0.11 |
| FGF2 | FGF2 Fibroblast growth factor 2 (basic) | 2.95 | 0.30 |
| FGFR1 | Fibroblast growth factor receptor 1 | 2.96 | 0.10 |
| FN1 | Fibronectin 1 | 2.96 | 0.35 |
| GDF10 | Growth differentiation factor 10 | 2.72 | 0.48 |
| GLI1 | GLI family zinc finger 1 | 3.30 | 0.20 |
| IGF2 | IGF2 Insulin-like growth factor 2 (somatomedin A) | 3.10 | 0.15 |
| ITGA3 | ITGA3 Integrin, alpha 3 | 3.18 | 0.23 |
| MMP2 | MMP2 | 4.43 | 1.06 |
| MMP9 | MMP9 | -3,41 | 0.19 |
| SERPINH1 | SERPINH1 | 2.93 | 0.16 |
| SMAD5 | SMAD5 | 2.53 | 0.52 |
| SP7 | Osterix | 3.01 | 0.16 |
| TGFBR2 | TGFBR2 | 2.54 | 0.45 |
| TWIST1 | TWIST1 | -6.89 | 0.17 |
| VDR | VDR | 2.523 | 0.47 |
| VEGFB | VEGFB | -6.487 | 0.19 |
